# Mobilizing the World Café Method for Adequate Development of Non-Technical Skills of Midwives in Morocco: A Pilot Experiment

**DOI:** 10.3390/healthcare11040519

**Published:** 2023-02-09

**Authors:** Asmaa Ghafili, Widad Azzouzi, Meryem Hamdoune, Abdellah Gantare, Claire Lobet-Maris, Maximilien Gourdin

**Affiliations:** 1 Nursing and Midwifery Unit, Laboratory of Health Sciences and Technologies, Higher Institute of Health Sciences, Hassan First University of Settat, Settat 26000, Morocco; 2 Institut de Recherche Expérimentale et Clinique, Pôle de Médecine Aiguë, Université Catholique de Louvain, 1200 Woluwe-Saint-Lambert, Belgium; 3 Royal Academy of Science, Letters and Fine Arts of Belgium, BE1000 Bruxelles, Belgium; 4 Technology, Ethics & Society Research Unit, Research Centre in Information, Law and Society (CRIDS), Namur Digital Institute (NADI), Faculty of Computer Sciences, University of Namur, 5000 Namur, Belgium; 5 Department of Anesthesiology, UCLouvain, CHU UCL Namur, 5530 Namur, Belgium

**Keywords:** non-technical skills, midwifery, World Café method, POCI model, continuing education

## Abstract

This article explores the development of the most critical soft skills in midwifery through the use of a participatory method called the World Café in the context of continuing education at the Formation and Simulation Center (FORSim) in Settat, Morocco. Non-technical skills include a set of metacognitive abilities that complement technical skills to ensure the safe execution of technical activities and the parturient’s satisfaction. In order to develop these midwifery skills through the World Café method, we invited nine midwives from two maternity units in the Casablanca-Settat region, with whom we elaborated our psychological, organizational, cognitive, and interactional (POCI) model. The study took place over a full day, structured into three distinct steps: a self-assessment of the level of mastery of the eight soft skills in the POCI model, four cycles of the World Café and, finally, a discussion of and feedback about the method. The use of the World Café method allowed for a dialogue on the possibilities of managing and addressing issues related to non-technical skills among midwives from various hospital settings. Based on the results, we found that the participants enjoyed the non-stressful atmosphere of the World Café and were very productive. The assessments and feedback from the midwives participating in this study suggest that managers can adopt the World Café approach to develop non-technical skills and enhance midwives’ interactions and soft skills as part of their continuing education.

## 1. Introduction

Non-technical skills (NTS) are described in the literature as a combination of interpersonal and social skills that complement technical skills and contribute to safe and effective technical performance [1].

Specifically, NTS are defined by Rhona Flin et al. [2] as “cognitive, social, and personal skills that complement technical skills and contribute to safe and effective task performance.” In 2015, Flin et al. [3] clearly demonstrated the importance of NTS, as poor soft skills can increase the risk of error, which in turn can increase the risk of an adverse event. For example, a Canadian study showed that the introduction of team briefings improved clinical practice [4]. Another study showed that the use of checklists reduced the number of communication errors and improved intra-team communication [5].

Based on an empirical study [6] conducted on the identification of critical NTS among midwives in the Casablanca-Settat region, we developed a model categorizing these NTS into four dimensions, each of which includes two critical soft skills for midwives. We called this model POCI:

- P: psychological = motivation and stress management.
- O: organizational = teamwork and conflict management.
- C: cognitive = adapting to complex situations and solving problems.
- I: interactional = communication and respect for privacy.

The choice of the World Café method for our intervention research echoes the work of Bazilio et al. [7], which highlighted the value of the method in nursing for creating a robust environment for strategic dialogue, multi-stakeholder engagement, multi-generational collaboration and collaborative-action design around issues facing advanced practice nurses [8].

The method, which focuses on collaborative intelligence, is centered on the cross-pollination of knowledge, avoiding the valuation of only the point of view or experience of a particular individual in discussions, as is the case in more directive structures such as structured interviews and focus groups.

This article focuses on the development of NTS among midwives. In this regard, we propose to provide feedback on the World Café method, which we applied experimentally with a group of midwives in order to develop their soft skills. Moreover, this is an opportunity for us to test the empirical validity of our modeling by applying World Café to the continuous training of midwives in order to develop the various soft skills identified primarily at the field level of our research.

## 2. Materials and Methods

**Figure 1:**
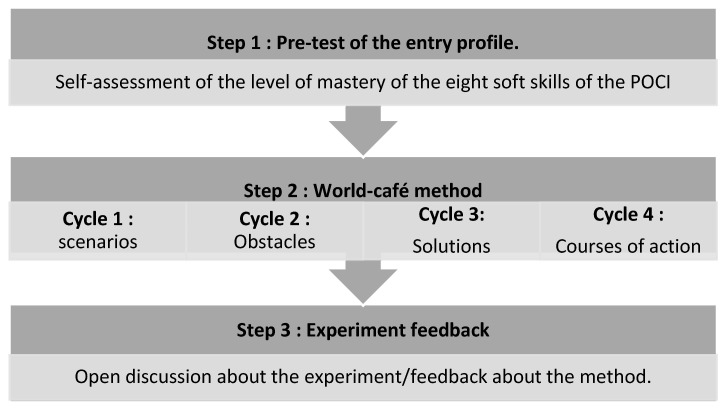
The three main steps of the World Café protocol.

The World Café method was applied with nine midwives from two maternity units in the Casablanca-Settat region. The research received the approval of the Moroccan Association for Research and Ethics (04/REC/22). The midwives were given both written and oral information about the study and they were invited to participate in a volunteer capacity. The research took place over a full day, structured into three distinct steps (Figure 1):

Pre-test: At the beginning of the session, we asked the nine midwives to complete a self-assessment form on their level of mastery of the key eight competencies in the POCI model to target those that needed to be strengthened.

We structured our World Café method into four cycles (Table 1):

**Feedback**: This last step concerns the post evaluation of the World Café method as a means of developing the non-technical skills of the POCI model within the framework of midwives continuing their education.

## 3. Results

**Step 1:** Pre-test of the entry profile.

First, we asked the nine midwives to complete a self-assessment form on their level of mastery of the POCI model’s non-technical skills, in order to identify those that required priority development for this group of midwives.

- The level of mastery of soft skills or NTS: MV = midwife

1: very poorly developed, 2: poorly developed, 3: acceptable, 4: mastered, 5: high mastery.

A: communication, B: respect for privacy, C: teamwork, D: conflict management, E: motivation, F: stress management, G: problem-solving, H: adaptation to complex situations.

As can be seen in the table above (Table 2), the least-developed skills among the participants were conflict management (44.44% of participants), stress management (50% of participants), and coping with complex situations (55.55% of participants).

**Step 2:** The World Café process

**Figure 2:**
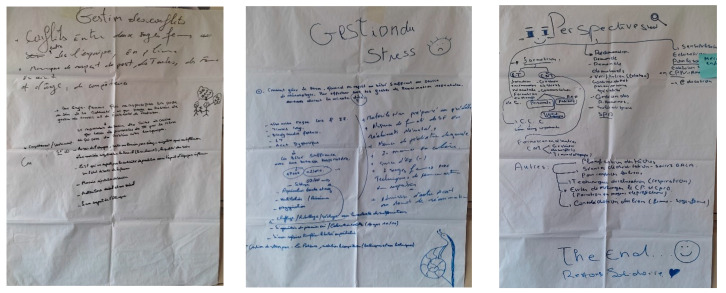
Scenario for the NTS of conflict- and stress-management, and the action plan for stress management.

In the first cycle, after splitting the nine midwives over three tables, we asked them to co-construct a scenario for each of the three non-technical skills targeted by the session. We invited the participants to change tables in order to progressively feed and consolidate the initial scenarios. In a second cycle, using the same process, we asked the midwives to identify the obstacles and difficulties encountered in each situation. During the third cycle, the participants were asked to reflect on solutions centered on the NTS targeted in each scenario. During the fourth and last cycle, the midwives were asked to reflect on the courses of action that could be taken to develop the NTS in order to avoid problems in the future. Table 3 illustrates the outputs of the nine midwives on each cycle of the World Café:

The images below (Figure 2) provide an idea of the dynamics of co-construction and discussion among the midwives traced on flip charts, which were later displayed while one member of each group summarized the main points.

On these flip charts (Figure 2), it can be observed that the participants were able to express their points in a comfortable and appropriate form, which facilitated the exchange between them.

**Step 3:** Experiment Feedback

This last step in our protocol was devoted to the feedback of the nine midwives, whom we recorded in order to analyze their statements subsequently, using the ATLAS.ti software.

**Figure 3:**
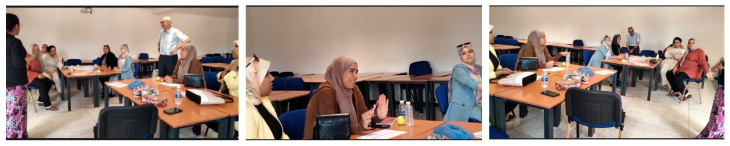
Pictures of the participants during the experiment.

Below are some live pictures (Figure 3) of the participants during their journey:

To conduct the analysis above, we adopted an approach advocated by Pierre Paillé [9], which is a reworked version of Glaser and Strauss’s [10] grounded theory. Since its elaboration, the grounded theory has undergone significant development all over the world, and still represents a solid reference that is often applied in qualitative research. The qualitative approach of Paillé is refines the conceptual apparatus of grounded theory. For example, Paillé proposes the concept of “theorization” instead of “theory” and “modeling” or “modeling test” instead of “model.”

These modifications are widely used within the research community and largely correspond to the objectives of our research, in the sense that the model that emanated from our research in the field was a “modeling test,” a model that is perfectible and under construction.

Regarding the use of the Atlas.ti software, the latter constitutes a computer solution similar to other common types of reflective-labeling software, such as NVivo, MAXQDA and HyperRESEARCH, which are used to support research through grounded theory.

This process involves the following stages:

(1) Phenomenological examination of the data.
(2) Analysis using conceptualizing categories.
(3) The linking of categories and properties.

It is then necessary to emphasize the links between the phenomena, the bond, the affinities, and the antagonisms.

(4) The analytical integration of the whole.
(5) The modeling of the emerging phenomena.
(6) The consolidation of the theorization.

The phenomenological examination of the data, the analysis using the conceptualizing categories, and the linking of the categories and properties through the Atlas.ti software resulted in four main categories:

- Midwives’ performance during the experiment.
- The NTS developed.
- The contributions of the method used.
- Implications for practice.

The analytical integration of the entire set brought out a central phenomenon: learning.

**Figure 4:**
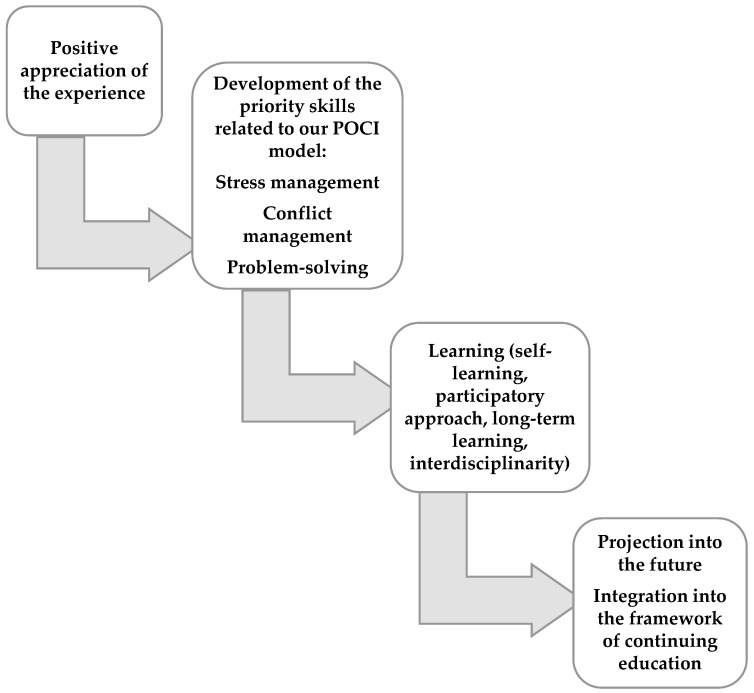
The modeling of the emerging phenomena.

The modeling of the emerging phenomena took the following form (Figure 4):

## 4. Discussion

The purpose of this study was to evaluate the World Café method as a methodology for developing non-technical skills. This objective is related to the broader research question of how to develop these skills in midwives in the context of their continuous education.

As a first step, we identified the most critical soft skills in midwifery practice through a field study, in which we met 30 midwives [6]. As a result, we developed our POCI model, which included the most important skills for a practicing midwife in order to guarantee good quality of care and high-performance service. Before proceeding to the implementation of this model in a health-care team, we performed a pilot experiment in order to both refine the model and, subsequently, test different participatory learning methods that can be proposed to staff in order to develop their non-technical skills, including the World Café method. Of the thirty midwives, nine responded affirmatively to our request to participate in this pilot experiment.

Our work is part of a research intervention. According to Claire Duchesne and Rodney Leurebourg [11], intervention research, as its name indicates, constitutes both a research method and a field-intervention approach whose objective is to produce knowledge that can be used to combine action and transformation. It consists of a variant of action research (AR), according to which the influence of the group encourages changes in individual attitudes and behaviors. From this perspective, the researcher is no longer simply an observer of a phenomenon, but intervenes in the action, in the research, and in the training of the participants.

The King Baudouin Foundation (2006) [12] defines the World Café method as a creative process that aims to facilitate constructive dialogue and the sharing of knowledge and ideas, with a view to creating a network of exchange and action. The process mimics a café setting, in which participants discuss an issue or topic in small groups around tables. At regular intervals, participants change tables.

The World Café relies on collective intelligence to identify and harness skills and competencies, as it allows participants to use logical thinking and focus on both adopting new perspectives to maximize the impact of their discussion and, ultimately, share their collective discoveries. The discussion is based on asking questions, encouraging members to share their point of view, and listening to others to examine the context and problem from multiple perspectives. Thus, participants plan group activities based on collective intelligence [13,14].

The literature shows that there have been no studies similar to ours, based on the testing of the World Café method as a tool for developing soft skills in midwives. However, we have consulted some research that encourages the adoption of this participatory method in the training of health personnel or professionals [15].

The use of the World Café method allowed a dialogue on the possibilities of managing and addressing issues related to non-technical skills among midwives from various hospital settings. Based on the results, we found that the participants enjoyed the non-stressful atmosphere of the World Café and were very productive. These observations echo those in a study conducted with nursing-student mentors. In that study, the participants explain was how the café environment helped them share ideas, develop opportunities to support student learning, and improve communication between the education department and the mentors [16].

According to our results, the World Café is considered as a particular method of education and participatory practice. The method is recognized for its successful contribution to the ability of staff to work in teams that consist of both expert staff and beginners, enhancing their skills to ensure comprehensive care (technical) and help in the development of health professionals (non-technical). In a manner that is consistent with the findings of this study, the World Café is an example of true learning, with midwives participating in the management of real-world problems, through teamwork, in an off-duty setting [17].

The teams recorded their discussions (scenarios, barriers, solutions, and perspectives) on flip charts, which were then posted while a member of each team summarized the main points. This method allowed a better understanding of the ideas that were most likely to help the midwives achieve their main goal of developing their non-technical skills in practice. The session took place in an atmosphere that was made comfortable by coffee breaks and lunch. Emee and Tonic [18] emphasize the importance of this comfortable and relaxing atmosphere to encourage participants to engage in reflection and participate in dialogue.

The midwives were divided into teams of three, in which they discussed their ideas and shared them with each other. They noted in the post-test phase that this structure allowed them to engage in dialogue and participate without any fear of being judged by others. They also noted that working in a small group was less intimidating and allowed them to feel comfortable expressing and sharing their ideas. Anderson (2011) shows the importance of working in very small groups to maximize the opportunity for deep learning [19].

According to a study based on the use of the World Café method in order to explore the professional journeys of some nurse educators, this method can be used effectively as a teaching tool to encourage nursing students to engage in the further exploration of different interests while developing communication, relationship-development, and collaborative-learning skills [20]. Regarding this topic, we observed that the participating midwives, through the method of the World Café, were able to exchange ideas with each other and maintain a communicative environment.

Following a study by Lohr et al. (2020), the WC process enabled a broad discussion of the research issue, which resulted in the generation of a variety of ideas. This is a useful way of accessing research, as it can bring out the main issues and themes that are important to a group [21]. As we observed in our experience, the midwives were able to discuss the problems and conflicts that most frequently occurred in the maternity department and, through this exchange, they devised some new ideas to help then overcome these situations.

Based on the World Café method, Kavanagh et al. [22] highlighted various needs among pharmacists in terms of interprofessional education and the importance of cooperation with other health professionals in research. This leads to the development of respect and an understanding of the different roles and competencies of the different professions, which provides a solid basis for interprofessional collaborative work and research. On the other hand, the midwives, during this experiment and throughout the scenarios, articulated the importance of continuing their education and the role of strengthening non-technical skills in ensuring quality care, high-performance services, and maintaining interpersonal relationships with colleagues.

The midwives particularly appreciated the fact that they co-constructed the scenarios, that it was they who, through discussion, identified obstacles and proposed solutions. They emphasized that this method allowed them to reflect more easily on the importance of non-technical skills because the situations dealt with were realistic and more rooted in their practices and ordinary work contexts. They also noted that this more realistic and contextualized approach allowed them to easily activate their collective intelligence to propose solutions based on an in-depth approach to the obstacles encountered in each situation. Furthermore, they stressed, following Charlotte Pace’s observations [23], the importance of motivation for them to be seen as able to manage their own work practices and to develop contextually and personally relevant strategies to help them improve the quality of their work. Finally, they underscored the fact that participation in a World Café not only allowed them to reflect on the importance of soft skills, discuss them, and work on them together, but also to practice and develop them throughout the exercise. This was particularly true for communication skills, interaction management, and team collaboration.

We observed from the feedback and the results of the constructions that the World Café method is indeed a tool for reflexivity and learning, as well as for the development of the three NTS, but a question arises as to whether all NTS be developed with the same method. To answer this question, the next work will be based on another reflexive-learning method in order to consolidate the transferability of our POCI model while diversifying the tested methods.

### 4.1. Limitations

The main purpose of our work is to report on a pilot experiment with a few midwives in order to determine the extent to which the World Café method can be used to develop non-technical skills. At this stage in our research, we do not claim that our results are statistically valid and generalizable. The results obtained within the framework of this pilot experiment will allow us only to test the possibility of implementing and refining our POCI model and to determine the extent to which we could transfer our discoveries to other situations and larger teams, in the context of multicenter research.

### 4.2. Future Approaches

Strengthen soft skills in more groups and with more midwives; present the tool and results to managers and invite them to continue using the method in their units; evaluate adherence to the recommendations resulting from this work in the services after 6 to 12 months; involve other groups to ensure reproducibility.

## 5. Conclusions

The evaluation and feedback from the midwives participating in this study showed that the World Café method can be adopted by managers in order to develop non-technical skills and strengthen interactions and communicative practices among midwives in the context of their continuous education. On the other hand, the World Café is, on the basis of our experience, a methodological tool that is fully consistent with the framework of intervention research. Indeed, the midwives emphasized during the evaluation of the session that this method not only allowed them to reflect together on their practices and find solutions to improve their NTS, but also had a transformative effect on them. In particular, they pointed out the effect of the World Café on their communication skills, as well as on their organizational skills in terms of teamwork management, and on their use of their cognitive skills in the collective resolution of complex situations.

## Figures and Tables

**Table 1:** The four cycles of World Café method.

World-Café Method			
Cycle 1	Cycle 2	Cycle 3	Cycle 4
The co-construction of a scenario that reflects a real situation, an undesirable event based on their experiences in the field.	The barriers associated with this situation.	Solutions to the specific situation.	The perspectives and courses of action to be adopted/recommended in terms of the development of the NTS are categorized by a table and by scenario.

**Table 2:** The levels of mastery for the eight non-technical skills among the nine midwives.

	MV1	MV2	MV3	MV4	MV5	MV6	MV7	MV8	MV9
A	2	5	5	2	3	5	5	4	3
B	4	5	5	5	3	5	4	5	5
C	5	4	4	4	4	5	4	5	5
D	4	4	4	3	2	5	4	3	3
E	3	5	4	4	3	5	5	4	4
F	4	5	4	3	1	5	3	3	2
G	5	5	4	4	1	5	4	3	3
H	5	5	4	3	2	5	3	3	3

**Table 3:** The four cycles of the World Café protocol.

World Café			
	Conflict Management	Stress Management	Adaptation to Complex Situations
**Cycle 1**	The conflict between two midwives during the change in shifts in the delivery room was due to the lack of respect for the post, the tasks, etc.	Reaction to newborn suffering in the neonatology department; the absence of an on-call pediatrician; the mother was very stressed and agitated; non-functional O²source; the midwife was on her own.	Admission of a woman to a birthing center, with complete dilatation and fetal-pelvic disappearance = transfer or operationn. However, there was no gynecologist, no fuel in the ambulance, and no feedback from those in charge. The family members were angry and asked for interventions as soon as possible, showing verbal aggression towards the midwives.
**Cycle 2**	– Problems with transmitting instructions. – Communication problems. – Age difference (generational conflict). – Lack of self-control. – Failure to respect professional ethics. – Disruption of the service.	Regarding the midwife: – Work overload. – Lack of task allocation. Concerning the service: – Lack of materials and responsible staff in neonatal reanimation. – Absence of a pediatrician on night shift.	– Lack of communication. – Poor management of resources. – Unmonitored pregnancy. – Stress and fear. – Family’s refusal to transfer the woman. – No regulation of the midwife’s function.
**Cycle 3**	– Transferring instructions between teams. – Mediation. – A discussion between the parties involved. – Designation of a leader from each team to convey the information. – Staff not assigned to fixed teams.	The service: – Staff assignment, and more efficient division of labor. The population: Awareness The staff: – Prioritizing the patient’s interests, accelerating resuscitative actions, asking for help, and ensuring relational connections.	– Explain the situation to the family to convince them to transfer the woman. – Complete a referral form. – Prepare the woman for the transfer. – Reassure the woman and accompany her to reduce stress. – File a report with the head of the service.
**Cycle 4**	– Continuing education. – Avoid confusion between professional and personal conflicts. – Regular meetings between midwives. – A clear protocol.	– Continuous training. – Task planning. – Meditation sessions and bonding evenings. – Staff motivation through recognition, and organizing days to celebrate midwifery.	– Continuous training. – Elaboration of job descriptions. – Organization of regular meetings to discuss problems. – Ability to manage stress and self-control. – Good management of resources.

## Data Availability

Data are available from the corresponding author upon reasonable request.
